# Simultaneous Inhibition of Glycolysis and Oxidative Phosphorylation Triggers a Multi-Fold Increase in Secretion of Exosomes: Possible Role of 2′3′-cAMP

**DOI:** 10.1038/s41598-020-63658-5

**Published:** 2020-04-24

**Authors:** Nils Ludwig, Saigopalakrishna S. Yerneni, Elizabeth V. Menshikova, Delbert G. Gillespie, Edwin K. Jackson, Theresa L. Whiteside

**Affiliations:** 1 Department of Pathology, University of Pittsburgh School of Medicine, Pittsburgh, PA 15213 USA; 2 UPMC Hillman Cancer Center, Pittsburgh, PA 15213 USA; 3 Department of Biomedical Engineering, Carnegie Mellon University, Pittsburgh, PA USA; 4 Department of Pharmacology and Chemical Biology, University of Pittsburgh School of Medicine, Pittsburgh, PA USA; 5 Departments of Immunology and Otolaryngology, Pittsburgh, PA 15213 USA

**Keywords:** Cancer metabolism, Tumour biomarkers, Tumour-suppressor proteins

## Abstract

Exosome secretion by cells is a complex, poorly understood process. Studies of exosomes would be facilitated by a method for increasing their production and release. Here, we present a method for stimulating the secretion of exosomes. Cultured cells were treated or not with sodium iodoacetate (IAA; glycolysis inhibitor) plus 2,4-dinitrophenol (DNP; oxidative phosphorylation inhibitor). Exosomes were isolated by size-exclusion chromatography and their morphology, size, concentration, cargo components and functional activity were compared. IAA/DNP treatment (up to 10 µM each) was non-toxic and resulted in a 3 to 16-fold increase in exosome secretion. Exosomes from IAA/DNP-treated or untreated cells had similar biological properties and functional effects on endothelial cells (SVEC4-10). IAA/DNP increased exosome secretion from mouse organ cultures, and *in vivo* injections enhanced the levels of circulating exosomes. IAA/DNP decreased ATP levels (p < 0.05) in cells. A cell membrane-permeable form of 2′,3′-cAMP and 3′-AMP mimicked the potentiating effects of IAA/DNP on exosome secretion. In cells lacking 2′,3′-cyclic nucleotide 3′-phosphodiesterase (CNPase; an enzyme that metabolizes 2′,3′-cAMP into 2′- and 3′-AMP), effects of IAA/DNP on exosome secretion were enhanced. The IAA/DNP combination is a powerful stimulator of exosome secretion, and these stimulatory effects are, in part, mediated by intracellular 2′,3′-cAMP.

## Introduction

Extracellular vesicles (EVs), including the small subset of EVs referred to as exosomes which are derived from the endocytic compartment of parental cells and range in size from 30 to 150 nm, play an important role in intercellular communication. The biogenesis of exosomes is distinct from that of other EVs, such as microvesicles (MVs) or apoptotic bodies^1^. It begins with the internalization of cell surface proteins by endocytosis and the sequestration of these proteins by early endosomes. In late endosomes, a process of reverse vesicular invagination leads to the formation of multivesicular bodies (MVBs), which are filled with numerous vesicles. Importantly the topography of proteins decorating these vesicles mimics that of the surface membrane of a parental cell^2^. Thus, exosomes differ from other EVs in that their vesicular cargo is derived from the proteins processed in late endosomes and packaged into vesicles in the MVBs. When MVBs fuse with the cell membrane of the parent cell, exosomes are released into the extracellular space^3^. Exosome packaging and their cellular secretion have been extensively investigated and while the general mechanistic underpinning their formation are described, it remains unclear to which extent the packaged and secreted exosomes are molecular mimics of their parental cells or whether they carry addressed instructions to the potential recipient cells^4^. Nevertheless, secreted and circulating exosomes are looked upon as a liquid biopsy, and their characteristics, cargos of proteins and nucleic acids and their identity with the parent cells have been of great interest.

Exosomes are in demand not only as potential non-invasive biomarkers but also as a delivery system of messages that can be transferred to or incorporated into the exosome cargo^5^. Compared to other nano-sized delivery systems, such as lipids, polymers, gold and silica materials, exosomes are living-cell derived, highly biocompatible nano-carriers with an intrinsic payload that can be experimentally modified^6^. Exosomes are characterized by much greater flexibility in loading desired antigens for effective delivery than are, e.g, liposomes^7^. Exosomes are reported to remain in the circulation longer than liposomes^8^, and they interact with a broad variety of cell targets, including dendritic cells (DCs)^9^. Exosome cargos avoid degradation or loss during delivery to distant sites^10^. Due to these characteristics, exosomes are considered to be favourable component of vaccines. However, the development of exosome-based vaccines, as well as other applications of exosomes, has been hindered by substantial technical difficulties in obtaining immunogenic exosomes that are free of plasma-derived or cell supernatant-derived “contaminants” in quantities adequate for *ex vivo* studies.

Although all cells secrete exosomes, there are substantial differences between cells in levels of released exosomes. Specifically, cultured tumor cells secrete enlarged quantities of exosomes, while normal tissue cells secrete small- to-moderate quantities^11^.

There is a need for standardizing and optimizing exosome production by cultured cell lines, and while we have previously described methods for achieving such optimization^11,12^, here we report a newly developed procedure for increasing exosome production *in vitro* and *in vivo* using a combination of two biochemical agents, sodium iodoacetate (IAA; glycolysis inhibitor) and 2,4-dinitrophenol (DNP; oxidative phosphorylation inhibitor).

## Results

### IAA/DNP stimulates exosome secretion *in vitro*

**Figure 1:**
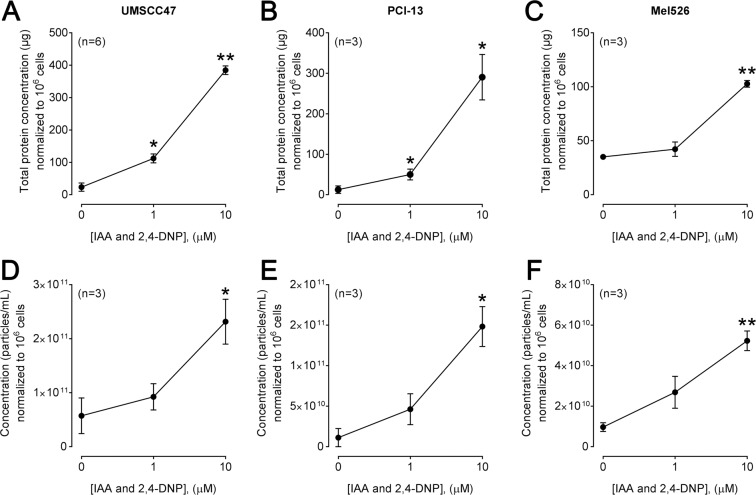
Exosome production in response to IAA/DNP. Levels of total exosomal protein in μg normalized to 10^6^ cells derived from UMSCC47 **(A)**, PCI-13 **(B)** or Mel526 **(C)** cell lines cultured in absence or presence (1 or 10 µM) of IAA/DNP. Results were validated by qNano for UMSCC47 **(D)**, PCI-13 **(E)** and Mel526 **(F)** and expressed as particle concentrations normalized to 10^6^ cells. Values represent means ± SEM; *p < 0.05; **p < 0.01.

**Figure 2:**
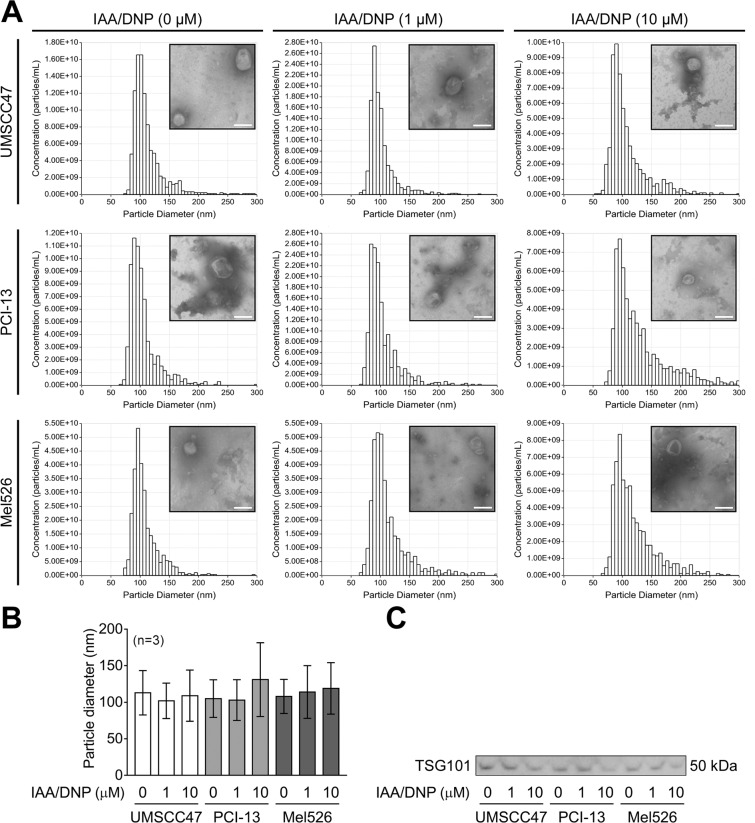
Properties of exosomes derived from cells treated with IAA/DNP. **(A)** TEM images of isolated and negatively-stained UMSCC47-, PCI-13- and Mel526-derived exosomes. Cells were treated with indicated concentrations of IAA/DNP. Uncropped TEM images can be found in supplementary information. **(A**,**B)** Size distributions of UMSCC47-, PCI-13- and Mel526-derived exosomes were measured by qNano. Values represent means ± SD. **(C)** Western blots of isolated UMSCC47- and PCI-13- and Mel526-derived exosomes with a TSG101 antibody. Full blots are presented in the supplementary information.

Exosomes were isolated from cancer cell lines UMSCC47, PCI-13 and MEL526, which were treated with 0, 1 or 10 µM IAA/DNP for 72 h. All cell cultures showed a concentration-dependent increase of exosomes in the conditioned medium quantified by BCA protein assays and expressed in µg as total exosomal protein as shown in Fig. 1A–C. Treatments of UMSCC47 cells with 10 µM IAA/DNP revealed a 3-fold increase of exosome secretion after 6 h, an almost 6-fold increase after 12 h and ≥10-fold increase for cellular treatments longer than 48 h. (Fig. S1). Results were validated by qNano, which measures particle size and concentrations (Fig. 1D–F). The particle size in fraction #4 of all three cell lines measured by qNano ranged from approximately 60 to 160 nm, and no alterations in exosome size were observed comparing exosomes derived from treated or untreated cells (Fig. 2A,B). Exosomes isolated from cells treated with increasing concentrations of IAA/DNP showed similar morphology by TEM (Fig. 2A). All exosomes carried TSG101, which indicated their origin from the endocytic compartment of the parent cell (Fig. 2C).

**Figure 3:**
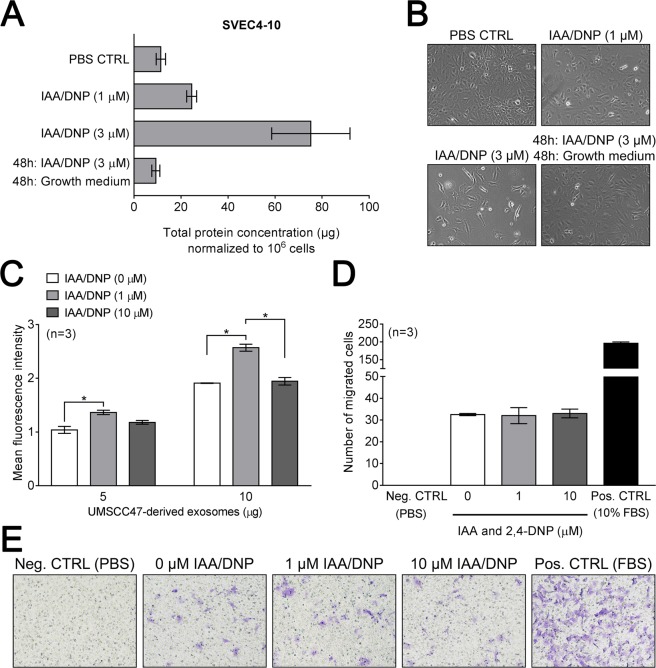
Functional activity of exosomes derived from cells treated with IAA/DNP. **(A)** Levels of total exosomal protein in μg normalized to 10^6^ cells derived from SVEC4-10 cells. **(B)** Representative images of SVEC4-10 cells treated with indicated concentrations of IAA/DNP. **(C)** Internalization of UMSCC47-derived exosomes by SVEC4-10 cells after 4 hours. Exosomes were derived from cells treated with the indicated concentrations of IAA/DNP. **(D)** Migration of SVEC4-10 cells towards serum-free media (Neg. CTRL), 10% FBS (Pos. CTRL) and 10 μg of protein of exosomes derived from cells treated with the indicated concentrations of IAA/DNP. **(E)** Representative images of migrated cells in 20x magnification. All values represent means ± SEM; *p < 0.05.

SVEC4-10 lympho-endothelial cells were used to confirm effects of IAA/DNP on normal (non-malignant) cells. IAA/DNP increased exosome secretion by SVEC4-10 in a concentration-dependent manner (Fig. 3A).

To measure biological activity of exosomes derived from treated or untreated cells, functional studies with SVEC4-10 lympho-endothelial cells were performed. Exosomes were internalized by SVEC4-10 cells within 4 h, and the same concentration of exosomes derived from treated or untreated cells had similar functional activity. SVEC4-10 cells internalized slightly more exosomes derived from cells treated with 1 µM IAA/DNP (Fig. 3C). The migration of SVEC4-10 cells was similarly stimulated by exosomes from treated or untreated cells (Fig. 3D,E).

### IAA/DNP combination stimulates exosome secretion ex vivo

**Figure 4:**
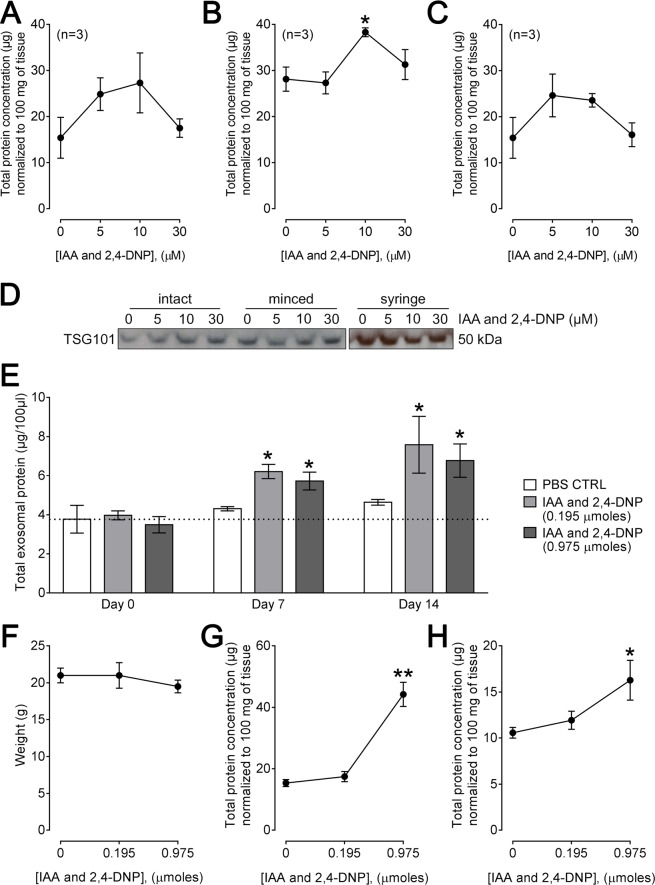
IAA/DNP stimulates exosome secretion *ex vivo* and increases circulating levels of exosomes *in vivo*. (**A–C**) Exosome production by tissue explants in response to IAA/DNP. Harvested kidneys were cultured for 48 hours with the indicated concentration of IAA/DNP. The tissues were cultured intact **(A)**, minced **(B)** or the treatment was injected into the intact tissue with a syringe **(C)**. Total protein concentrations are expressed in μg and were normalized to 100 mg of tissue. **(D)** Western blots of isolated exosomes derived from tissue explants with a TSG101 antibody. Full blots are presented in the supplementary information. **(E)** Plasma levels of total exosomal protein in μg normalized to 100 μl of plasma after 0, 7 and 14 days of treatment. Mice were either treated with PBS, 0.195 μmoles IAA/DNP or 0.975 μmoles of IAA/DNP. Dotted line indicates basal level of circulating exosomes. **(F)** Body weight (g) of mice after 14 days of treatment. **(G)** Levels of total exosomal protein in μg normalized to 100 mg of tissue derived from kidneys which were harvested after 14 days of treatment with indicated concentrations of IAA/DNP. **(H)** Levels of total exosomal protein in μg normalized to 100 mg of tissue derived from livers which were harvested after 14 days of treatment with indicated concentrations of IAA/DNP. All values represent means ± SEM; *p < 0.05; **p < 0.01.

To investigate further the stimulatory effects of IAA/DNP on exosome secretion, tissue explants (kidneys) were harvested from C57BL/6 mice and cultured for 48 h in the presence or absence of IAA/DNP. Some kidneys were minced and other kidneys were left intact. Intact kidneys also received injections of IAA/DNP at three sites using a syringe. IAA/DNP caused a concentration-dependent increase of exosome release from tissue explants into culture medium. The concentration of 10 µM IAA/DNP was found to be most effective for the intact and minced tissues, whereas the tissue explants treated with IAA/DNP injections already responded to 5 µM IAA/DNP (Fig. 4A–C). Similar TSG101 levels were detected in all exosome samples regardless of the concentration of IAA/DNP used (Fig. 4D).

### IAA/DNP stimulates exosome secretion in vivo

*In vitro* and *ex vivo* results were validated by injecting IAA/DNP into mice. Based on the amount of body fluid of mice, a dose of 0.195μmoles of IAA/DNP was used to provide an initial concentration of 10 μM in the body fluids. Another group of mice received a 5-fold higher dose (0.975μmoles). The injections did not affect the weight of the animals and did not alter their behaviour or induced signs of stress or pain (Fig. 4F). Both doses of IAA/DNP stimulated the levels of circulating exosomes in the blood compared to control mice (Fig. 4E). Kidneys and livers of mice were harvested and cultured for 48 h after 14 days of treatment with IAA/DNP. Notably, exosome levels were elevated in both tissue types in a dose-dependent manner (Fig. 4G,H).

### IAA/DNP causes a non-toxic energy depletion in cultured cells

The numbers of dead cells in the culture medium measured indirectly by LDH assays showed low levels of LDH in the culture medium up to concentrations of 10 µM IAA/DNP (Fig. S2). However, 15 µM IAA/DNP led to a very slight, but statistically significant (p = 0.033), increase in LDH. Therefore, 10 µM was used as the highest concentration of IAA/DNP in subsequent assays and was considered as a non-toxic dose.

**Figure 5:**
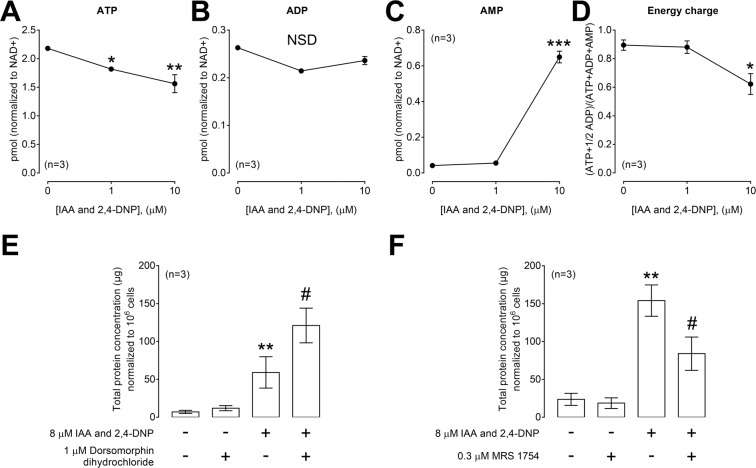
IAA/DNP causes energy depletion in cultured cells. Levels of ATP (**A**), ADP (**B**) and AMP (**C**) were quantitated by HPLC, and the data were corrected for cell number. (**D**) Based on the data shown in (**A**–**C**) the cellular energy charge was calculated using the indicated formula. (**E**) Exosome production in response to IAA/DNP in combination with dorsomorphin dihydrochloride. Levels of total exosomal protein in μg normalized to 10^6^ cells derived from UMSCC47 cells. (**F**) Exosome production in response to IAA/DNP in combination with MRS 1754. Levels of total exosomal protein in μg normalized to 10^6^ cells derived from UMSCC47 cells. Values represent means ± SEM; *p < 0.05 vs. untreated; **p < 0.01 vs. untreated; ***p < 0.001 vs. untreated; #p < 0.05 vs. IAA/DNP.

To further characterize the effects of IAA/DNP, HPLC was used to quantify levels of ATP, ADP and AMP after treatment of cells with 0, 1 and 10 µM IAA/DNP. Data were normalized to account for differences in cell number between conditions. In cultured cells, IAA/DNP decreased ATP levels (Fig. 5A) but increased AMP levels (Fig. 5C), and these effects were concentration dependent. ADP levels were not affected by IAA/DNP treatment (Fig. 5B). Calculating the energy status of the cells using the formula (ATP + 1/2 ADP)/(ATP + ADP + AMP) revealed a significant drop of the energy charge (Fig. 5D).

To test the toxicity of IAA/DNP, SVEC4-10 were cultured in the presence of IAA/DNP for 48 h followed by 48 h of culture in the regular growth medium. This led to exosome levels which were comparable to those in untreated cells, indicating that the treatment with IAA/DNP is reversible and non-toxic (Fig. 3A,B).

### Stimulation of exosome secretion by IAA/DNP is augmented by AMPK inhibition and attenuated by A_2B_R antagonism

Stimulation of exosome production by IAA/DNP was significantly augmented by an inhibitor (dorsomorphin) of AMP-activated protein kinase (AMPK) (p < 0.05, Fig. 5E).

In contrast, stimulation of exosome production by IAA/DNP was significantly decreased by the A_2B_R antagonist MRS 1754 (p < 0.05, Fig. 5F).

### 8-Br-2′3′-cAMP enhances exosome production, and the effects of both 8-Br-2′,3′-cAMP and IAA/DNP are augmented in CNPase knockout cells

**Figure 6:**
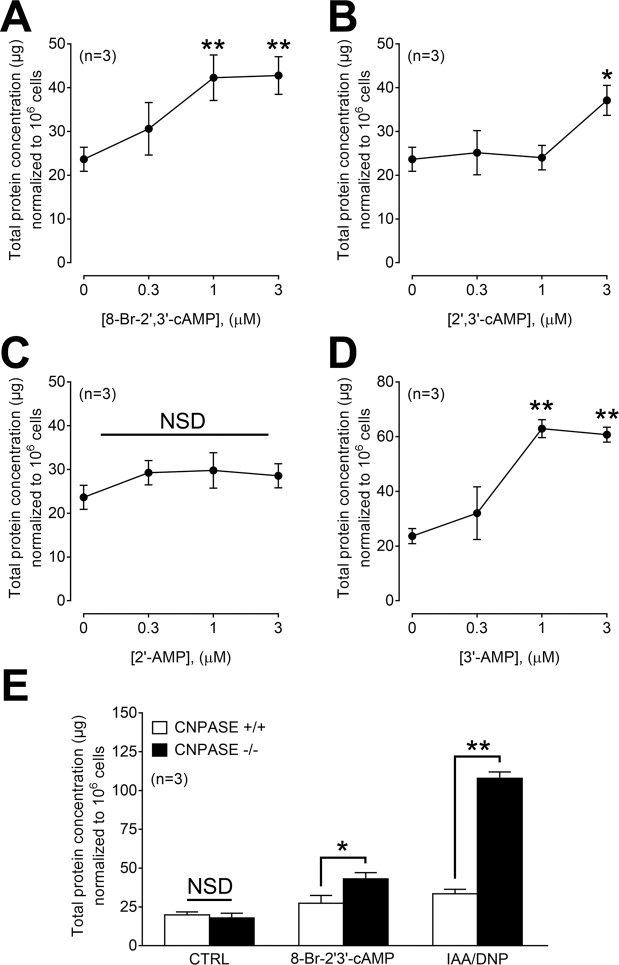
Exosome production by UMSCC47 cells in response to 8-Br-2′,3′;-cAMP (**A**), 2,′3′-cAMP (**B**), 2′-AMP (**C**) and 3′-AMP (**D**). Levels of total exosomal protein in μg normalized to 10^6^ cells. (**E**) Exosome production by PGVSMCs isolated from CNPASE+/+ and −/− rats in response to 8-Br-2′,3′-cAMP (0.3 µM) and IAA/DNP (5 µM). Values represent means ± SEM; *p < 0.05; **p < 0.01.

2′,3′-cAMP (not to be confused with the 2^nd^ messenger 3′,5′-cAMP) is a recently described endogenous non-canonical cyclic nucleotide, the production of which is stimulated by energy depletion with IAA/DNP in a concentration-dependent manner^13,14^. Extracellular 2′,3′-cAMP is metabolized to 2′-AMP and 3′-AMP, which in turn are metabolized to adenosine^14^. Importantly, intracellular 2′,3′-cAMP opens mitochondrial permeability transition pores (mPTPs^15^); and triggers stress granule formation^16^. Thus extracellular 2′,3′-cAMP can engage A_2B_R via adenosine, and intracellular 2′,3′-cAMP can compromise cellular energy production via opening mPTPs and stimulating the production of stress granules. Therefore, it is conceivable that the effects of IAA/DNP on exosome production are mediated in part by 2′,3′-cAMP. To test this, we examined the effects of a cell membrane permeable form of 2′,3′-cAMP, namely 8-Br-2′,3′-cAMP, on exosome production. As shown in Fig. 6A, 8-Br-2′3′-cAMP led to a concentration-dependent increase in exosome secretion (*p* < 0.01). Exogenous 2′3′-cAMP, which has very limited cell membrane permeability, also stimulated exosome production, but only at high concentrations (*p* < 0.05, Fig. 6B). Because extracellular 2′,3′-cAMP is metabolized to extracellular 2′-AMP and/or 3′-AMP, which in turn are converted to extracellular adenosine^14^, we also examined the effects of both exogenous 2′-AMP and 3′-AMP on exosome secretion. Although 2′-AMP did not affect exosome secretion (Fig. 6C), 3′-AMP significantly stimulated exosome release (*p* < 0.01, Fig. 6D). Because CNPase metabolizes intracellular 2′,3′-cAMP, we examined the release of exosomes induced by either 8-Br-2′,3′-cAMP or IAA/DNP in PGVSMCs obtained from CNPase+/+ versus CNPase −/− rats. Under basal conditions, exosome release was similar in CNPase+/+ versus CNPase −/− cells (Fig. 6E). In contrast, stimulation of exosome production in PGVSMCs by either 8-Br-2′3′-cAMP or IAA/DNP was greater in CNPase −/− compared with CNPase+**/+** cells (Fig. 6E; *p < 0.05 and **p < 0.01, respectively).

## Discussion

The clinical use of exosomes both as biomarkers of disease (e.g., cancer^17^, critical illness^18^ or cardiovascular diseases^19^) and carriers of drugs and biologics^5,20^ is of great current interest. One of the most crucial limitations to achieve clinical use is the purification of exosomes in sufficiently large quantities. Although isolation techniques are constantly improving and several methods have been suggested to stimulate exosome release^21^, the reported techniques only yield limited quantities of exosomes and indicate that the need for an exosome stimulant remains unmet.

Several reports in the literature describe conditions or agents, such as e.g., monensin^22^ or heat^23^, which increase exosome secretion by parental cells. In general, cells which are under stress are known to increase exosome secretion: thus, oxidative stress induced by ethanol increased exosome secretion by retinal pigment epithelium cells^24^ or glucose starvation enhanced exosome secretion in cardiomyocytes^25^. However, the effects of glucose deprivation on exosome release is not robust and cannot be used *in vivo*. Additionally, these reports did not evaluate mechanisms or provide adequate details of the increased exosome production. Nevertheless, by focusing on a potential connection between energy depletion by glucose starvation and exosome release, they provided a rationale for studying IAA/DNP. It has been reported that the combination of IAA and DNP induces a potent reduction of cellular energy charge by simultaneously blocking oxidative phosphorylation and glycolysis^26^. We reasoned that blocking cellular pathways of energy production using IAA and DNP might be an effective strategy for releasing exosomes. Indeed, as shown in this report, IAA/DNP is a powerful stimulator of exosome secretion in cultured cells and in animal models.

To the best of our knowledge, IAA/DNP is the most effective method yet discovered to stimulate exosome release, and in our hands, it is more efficacious than other commonly used stimulators of exosome secretion (Fig. S3). Datta et al. screened the effects of 4580 pharmacologically compounds on exosome release and only 6 were found to be activators of exosome biogenesis with forskolin being the most potent one (6-fold increase)^27^. Also, IAA/DNP is safe, can be used both *in vitro* and *in vivo* and works across a variety of cell lines. It might therefore accelerate exosome research and be used as a tool for the generation of exosomes in different settings. However, even though the IAA/DNP treatment increases exosome secretion and even if the effect of these exosomes does not change the cell migration assays, other differences (e.g., in composition, heterogeneity, functional effects in other potency assays) might hypothetically change and should be investigated in future studies. Also, the protein-based quantification of circulating exosomes in a complex biofluid, such as mouse plasma, may also detect co-isolated non-exosome associated proteins. In particular, LDL, VLDL and chylomicron contaminations have been reported and might contribute to the heterogeneity of exosomes isolated from plasma^28–30^.

**Figure 7:**
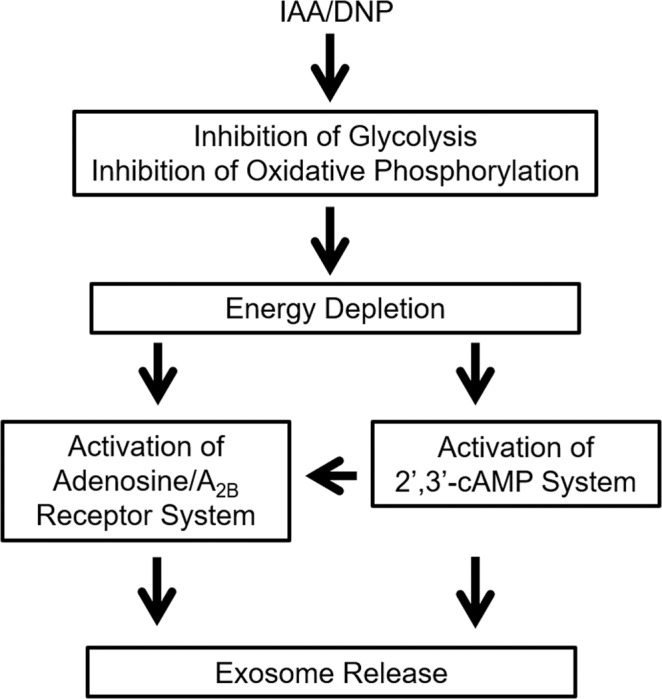
Schematic summarizes the biochemical steps in the stimulation of exosome release by IAA/DNP. The simultaneous inhibition of glycolysis and oxidative phosphorylation leads to energy depletion in the cells (decreased ATP levels, elevated AMP levels). As a result of the energy depletion, the cells release adenosine, which activates the A_2B_ receptor system which then enhances exosome release. Simultaneously, the cells release 2′,3′-cAMP, which stimulates the release of exosomes directly, but can also be a source for adenosine, which again can activate the adenosine receptor system.

The underlying mechanisms for the elevated exosome secretion after IAA/DNP treatment are summarized in Fig. 7. IAA inhibits glycolysis and DNP inhibits oxidative phosphorylation and, thereby, the combination severely suppresses energy charge. Decreased ATP and increased AMP levels in IAA/DNP-treated cells confirm energy depletion. Further, AMP accumulation is well known to trigger two processes: 1) activation of adenosine receptors (ARs) via adenosine production from AMP^14^, and 2) activation of AMPK^31^. Our experiments show that blocking A_2B_Rs attenuates the effects of IAA/DNP and blocking of AMPK augments IAA/DNP effects on exosome release. Therefore, we conclude that, in part, the ability of IAA/DNP to increase exosome release is mediated via A_2B_Rs; but likely activation of AMPK (known to enhance energy production) attenuates the effects of IAA/DNP. Because it is known that IAA/DNP combination increases 2′,3′-cAMP^13^, we examined the role of endogenous 2′,3′-cAMP in exosome release by using 8-Br-2′,3′-cAMP and by using CNPase knockout cells. These experiments confirmed that 2′,3′-cAMP plays a role in the IAA/DNP-mediated release of exosomes from cells. Although the mechanism by which 2′,3′-cAMP increases exosome release remains unknown, it could involve: 1) formation of adenosine^14^; 2) inhibition of mitochondrial function by opening mPTPs^15^; 3) formation of stress granules, which would block protein synthesis^16^; and 4) other “direct” effects of 2′,3′-cAMP on the process of exosome secretion. Besides activating the adenosine pathway and the 2′,3′-cAMP axis there may be other effects triggered by energy depletion induced by IAA/DNP. A recently published preprint by Frühbeis et al. describes further details and reports, that knockout of CNPase decreases basal release of exosomes from oligodendrocytes. They also reported that oligodendrocyte-derived exosomes are taken up by neurons and facilitate axonal transport. Notably, exosomes from CNPase knockout cells lack the ability to support nutrient deprived neurons and to promote axonal transport^32^.

IAA/DNP is the most effective method yet discovered to stimulate exosome release that involves, at least, A_2B_Rs and 2′,3′-cAMP. This method allows for a harvest of ample exosomes from various cells and may serve as a platform technology for the development of exosome-based therapies in the future.

## Materials and methods

### Chemicals

Sodium iodoacetate (IAA), 2.4-dinitrophenol (DNP), adenosine 2′,3′-cyclic monophosphate (2′,3′-cAMP), adenosine 2′-monophosphate (2′-AMP), adenosine 3′-monophosphate (3′-AMP) were from Sigma–Aldrich (St. Louis, MO). Dorsomorphin dihydrochloride and MRS 1754 were from Tocris Bioscience (Bristol, UK). 8-Bromoadenosine-2′3′-cyclic monophosphate (8-Br-2′3′-cAMP) was from BIOLOG (Bremen, Germany).

### Cell lines

Cells lines included in this study are listed in Table 1. All cell lines were grown at 37 °C in the atmosphere of 5% CO_2_ in air. Cultures were supplemented with fetal bovine serum (FBS) depleted of exosomes by ultracentrifugation at 100,000xg for 3 h. Cells were cultured in 150 cm^2^ cell culture flasks using 25 mL of culture medium. Media used for cell cultures are described in Table 1. Seeding protocol was optimized for each cell type as described by us^12^. After seeding, cells were allowed to attach to the flask for 6 h, were then treated with indicated reagents and incubated for 48 or 72 h as indicated.

**Table 1:** Cell lines included in this study^a^.

Cell lines	Cell type	Origin	Source	Media
UMSCC47	HPV(+) head and neck cancer cells	Dr. Thomas Carey (University of Michigan)	Robert L. Ferris (UPMC Hillman Cancer Center)	DMEM (Lonza Inc.), 1% (v/v) penicillin/streptomycin, 10% (v/v) exosome-depleted FBS (Gibco, Thermo Fisher Scientific)
PCI-13	HPV(−) head and neck cancer cells	Established and maintained in our laboratory^38^		DMEM, 1% (v/v) penicillin/streptomycin, 10% (v/v) exosome-depleted FBS
Mel526	Metastatic melanoma cells	Marincola *et al*.^39^	Walter J. Storkus (Department of Immunology, University of Pittsburgh)	RPMI-1640, 1% (v/v) penicillin/streptomycin, 10% (v/v) exosome-depleted FBS
SVEC4-10	Endothelial cells	O’Connell and Edidin^40^	ATCC, Manassas, VA, USA, cat. # CRL-2181	DMEM, 1% (v/v) penicillin/streptomycin, 10% (v/v) exosome-depleted FBS

^a^Cell lines included in this study and information about their type, origin and source as well as media used for their culture.

### Exosome isolation by mini-SEC

Processing of supernatants and exosome isolation by mini-SEC was performed as previously described^11^. Briefly, cell culture supernatants were centrifuged at room temperature (RT) for 10 min at 2000 × g, transferred to new tubes for centrifugation at 10,000 × g at 4 °C for 30 min and filtrated using a 0.22 µm bacterial filter. Afterwards, aliquots of supernatants were concentrated by using Vivacell 100 concentrators at 2000 × g. 1 mL of concentrated supernatant was loaded on a 10 cm-long Sepharose 2-B column and eluted with PBS, and individual 1 mL fractions were collected. Fraction #4 containing non-aggregated exosomes was used in subsequent assays. Our established isolation technique fulfils the criteria of the MISEV2018 guidelines and we therefore use the term ‘exosomes’ throughout the manuscript^33^.

### Protein concentration

Protein concentrations were determined using a BCA protein assay (Pierce Biotechnology, Rockford, IL, USA) according to the manufacturer’s instructions.

### Transmission electron microscopy (TEM)

TEM was performed as previously described^11^. Freshly isolated tumor-derived exosomes (TEX) or normal cell-derived exosomes were placed on copper grids coated with 0.125% Formvar in chloroform and stained with 1% (v/v) uranyl acetate in ddH2O. A JEM 1011 microscope was used for exosome visualization.

### Tunable resistive pulse sensing (TRPS)

Size distribution and concentrations of the particles in isolated exosome fractions were analyzed using tunable-resistive pulse sensing (TRPS) by qNano (Izon) as described previously^34^.

### Western blot analysis

To concentrate isolated exosomes, 0.5 mL 100 K Amicon Ultra centrifugal filters (EMD Millipore) were used for centrifugation at 4000 × g. Each lane was loaded with 5 μg of fraction #4 proteins, and PVDF membranes were incubated overnight at 4 °C with a TSG101 antibody (1:1000, ab30871, Abcam, Cambridge, MA) as previously described^34^.

### Cell migration

Cell migration by SVEC4-10 endothelial cells (ECs) was analyzed as previously described by us^12^. Briefly, 5 × 10^4^ SVEC4-10 cells were starved in serum-free media overnight and were added to the upper compartment of 24-well transwell plates with 8 µm pore diameter (Corning). Cells migrated towards serum-free medium or the medium supplemented with 10 µg exosomes derived from UMSCC47 cells treated with 0, 1 or 10 µM of IAA/DNP or 10% FBS, which were added to the lower compartment. After 6 h of incubation at 37 °C, non-migrating cells in the upper chamber were removed with cotton swabs. Migrating cells on the lower surface of the membrane were fixed in methanol and stained with 0.2% crystal violet (Sigma-Aldrich). The number of migrated cells was counted in a light microscope in six randomly selected regions of interest at 20x magnification using an Olympus BX51 microscope (Olympus America, Center Valley, PA).

### Uptake of exosomes by SVEC4-10 cells

5 × 10^3^ SVEC4-10 cells were seeded in 24-well plates and incubated for 24 h. 5 or 10 µg of TEX isolated from UMSCC47 cells treated with 0, 1 or 10 µM of IAA/DNP were labeled with SYTO RNASelect Green Fluorescent Cell Stain (Invitrogen) using manufacturer’s instructions and added to the cell culture for 4 h. Cells were washed twice with PBS and were harvested. Mean fluorescence intensity (MFI) of the cells was determined using a flow cytometer (Gallios; Beckman Coulter, Miami, FL, USA). Data were analyzed using Kaluza software (version 1.0; Beckman Coulter).

### Isolation of exosomes from tissue explants

Kidneys were harvested from 6 week old female C57BL/6 mice in an aseptic manner and immediately cultured in 6-well plates using 5 ml of DMEM supplemented with 1% (v/v) penicillin/streptomycin for 48 h as described by Mincheva-Nilsson *et al*.^35^. Tissue explants were treated with indicated concentrations of IAA/DNP. The treatment was given to intact tissue explants, minced tissue or was injected with an insulin syringe (29 G × 1/2″, Exelint, Redondo Beach, CA, USA) at three different locations. Supernatant was collected by gentle aspiration including washing of the tissue and the walls of the culture vessel. Processing of supernatants and exosome isolations were performed as described above.

### LDH assay

LDH release of cultured cells was performed using Pierce LDH Cytotoxicity Assay Kit (Thermo Scientific) following the manufacturer’s instructions. Cells were cultured for 72 h with indicated concentrations of IAA/DNP.

### Extraction and quantitation of NAD+, ATP, ADP, and AMP

ATP, ADP, and AMP were measured in cells treated with 0, 1 and 10 µM IAA/DNP using high performance liquid chromatography (HPLC). The protocol for the extraction of NAD+, ATP, ADP and AMP was previously described in detail^36^.

### Isolation of rat CNPASE+/+ and −/− PGVSMCs

2′,3′-Cyclic nucleotide 3′-phosphodiesterase (CNPase) knockout rats used in this investigation were generated by the MCW Gene Editing Rat Resource Program (Dr. Aron M. Geurts, Department of Physiology and Human Molecular Genetics Center, Medical College of Wisconsin, Milwaukee, WI). This strain was produced by injecting a CRISPR targeting the sequence GCTACTGCCGCCGGGACATC into rat embryos. The resulting mutation was a 7 base pair deletion in exon 2. Animals were genotyped by PCR. Preglomerular vascular smooth muscle cells (PGVSMCs) were isolated from kidneys of wild type (CNPase+/+) and knockout (CNPase −/−) rats using our previously described method^37^. Cells were cultured at 37 °C in the atmosphere of 5% CO_2_ in air using DMEM supplemented with exosome-depleted FBS. Exosome isolation was performed as described above.

### Animal study

This study was carried out in strict accordance with the recommendations in the Guide for the Care and Use of Laboratory Animals of the NIH. The protocol (18042580) was approved by the institutional Animal Care and Use Committee of the University of Pittsburgh (Animal Welfare Assurance Number: D16-00118). Female C57BL/6 mice aged 6 to 8 weeks were purchased from Jackson Laboratories. IAA/DNP was injected intraperitoneally daily for 14 days at the concentration of 0.195 or 0.975 μmoles per 100 µl. PBS injections of the same volume served as vehicle control. Blood was collected by submandibular bleeding on days 0, 7 and 14 of the experiment. Plasma was isolated by centrifuging at 1000xg for 10 min and further processed by spinning for 30 min at 10,000 × g and filtration with a 0.22 µm filter. Next, exosomes were isolated as described above. Additionally, kidneys and livers were harvested from all animals on day 14 and were cultured for 48 h. The supernatant of organ cultures was collected and exosomes were isolated as described above.

### Statistical analysis

All data were analysed using the GraphPad Prism software (v7.0). Values are expressed as means ± SD or SEM as indicated in the figure legends. Differences between groups were assessed by Student *t* test and differences were considered significant at *p* < 0.05.

## Supplementary information

Supplementary information.
