# Author Correction: Simultaneous Inhibition of Glycolysis and Oxidative Phosphorylation Triggers a Multi-Fold Increase in Secretion of Exosomes: Possible Role of 2′,3′-cAMP

**DOI:** 10.1038/s41598-020-70340-3

**Published:** 2020-08-17

**Authors:** Nils Ludwig, Saigopalakrishna S. Yerneni, Elizabeth V. Menshikova, Delbert G. Gillespie, Edwin K. Jackson, Theresa L. Whiteside

**Affiliations:** 1 Department of Pathology, University of Pittsburgh School of Medicine, Pittsburgh, PA 15213 USA; 2 UPMC Hillman Cancer Center, Pittsburgh, PA 15213 USA; 3 Department of Biomedical Engineering, Carnegie Mellon University, Pittsburgh, PA USA; 4 Department of Pharmacology and Chemical Biology, University of Pittsburgh School of Medicine, Pittsburgh, PA USA; 5 Departments of Immunology and Otolaryngology, Pittsburgh, PA 15213 USA

Correction to: *Scientific Reports* https://doi.org/10.1038/s41598-020-63658-5, published online 24 April 2020

The original version of this Article contained an error in the title of the paper, where the expression “2′,3′-cAMP” was incorrectly given as “2′3′-cAMP”.

The original version of this Article also contained errors in the Abstract.

“In cells lacking 2′,3′-cyclic nucleotide 3′-phosphodiesterase (CNPase; an enzyme that metabolizes 2′,3′-cAMP into 2′- and 3′-AMP), effects of IAA/DNP on exosome secretion were enhanced.”

now reads:

“In cells lacking 2′,3′-cyclic nucleotide 3′-phosphodiesterase (CNPase; an enzyme that metabolizes 2′,3′-cAMP into 2′-AMP), effects of IAA/DNP on exosome secretion were enhanced.”

These errors have now been corrected in the PDF and HTML versions of the Article and in the accompanying Supplementary Information file.

