# Supplementary material for: Simultaneous Inhibition of Glycolysis and Oxidative Phosphorylation Triggers a Multi-Fold Increase in Secretion of Exosomes: Possible Role of 2′3′-cAMP
Source: Sci Rep. 2020 Apr 24;10:6948. doi: 10.1038/s41598-020-63658-5 (PMC7181876; doi:10.1038/s41598-020-63658-5)
Supplement: Supplementary file 1 — Supplementary information. [file 41598_2020_63658_MOESM1_ESM.pdf]

Supplementary Information

Simultaneous Inhibition of Glycolysis and Oxidative Phosphorylation Triggers a Multi-Fold Increase in Secretion of Exosomes: Possible Role of 2'3'-cAMP

Nils Ludwig, Saigopalakrishna S. Yerneni, Elizabeth V. Menshikova,
Delbert G. Gillespie, Edwin K. Jackson and Theresa. L. Whiteside

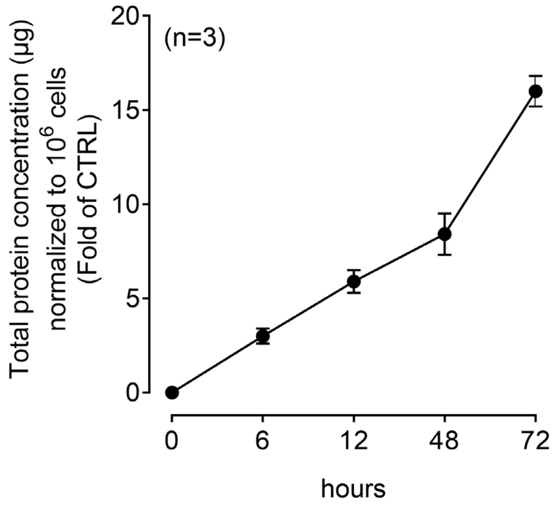

Figure S1: Time course of exosome production in response to 10 µM IAA/DNP. Levels of total exosomal protein in µg normalized to 10^6 cells derived from UMSCC47 were determined by BCA and normalized to untreated CTRL. Values represent means ± SEM.

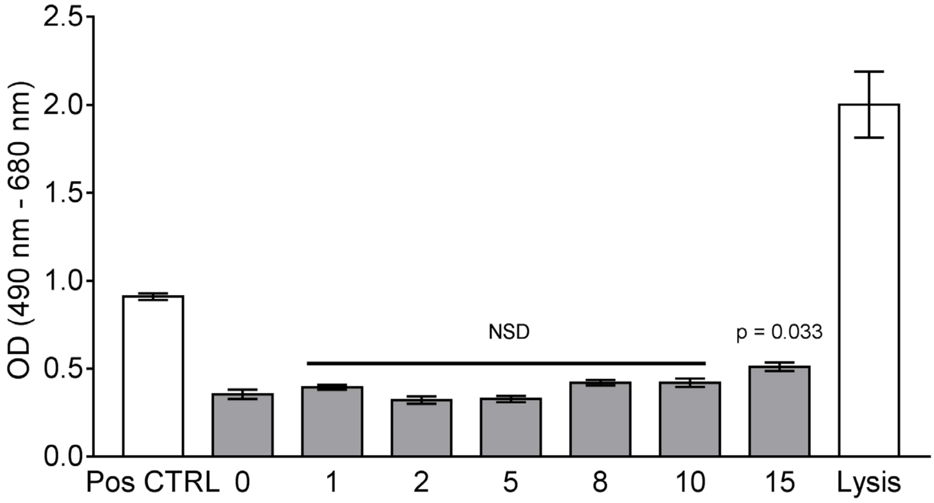

Figure S2: IAA/DNP causes non-toxic energy depletion in cultured cells. Culturing UMSCC47 cells in the presence of 1-10 µM IAA/DNP revealed steady levels of LDH release. IAA/DNP concentrations of 15 µM caused a statistically significant increase of LDH release compared to control ($p = 0.033$). Values represent means ± SEM.

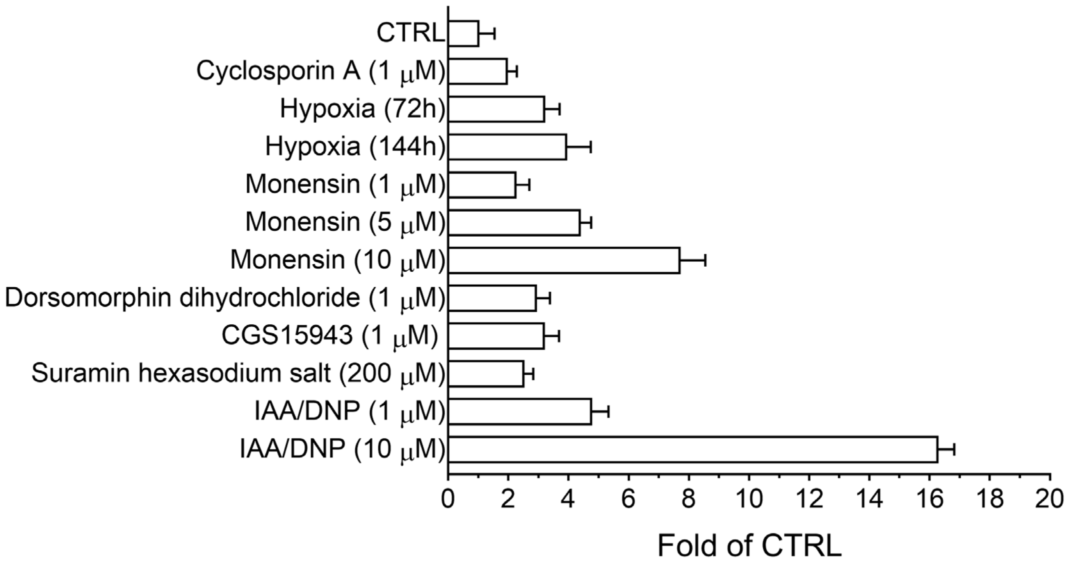

Figure S3. Exosome production in response to indicated treatments. Levels of total exosomal protein in μg normalized to 10^6 cells derived from UMSCC47 expressed as Fold of CTRL. Values represent means ± SEM.

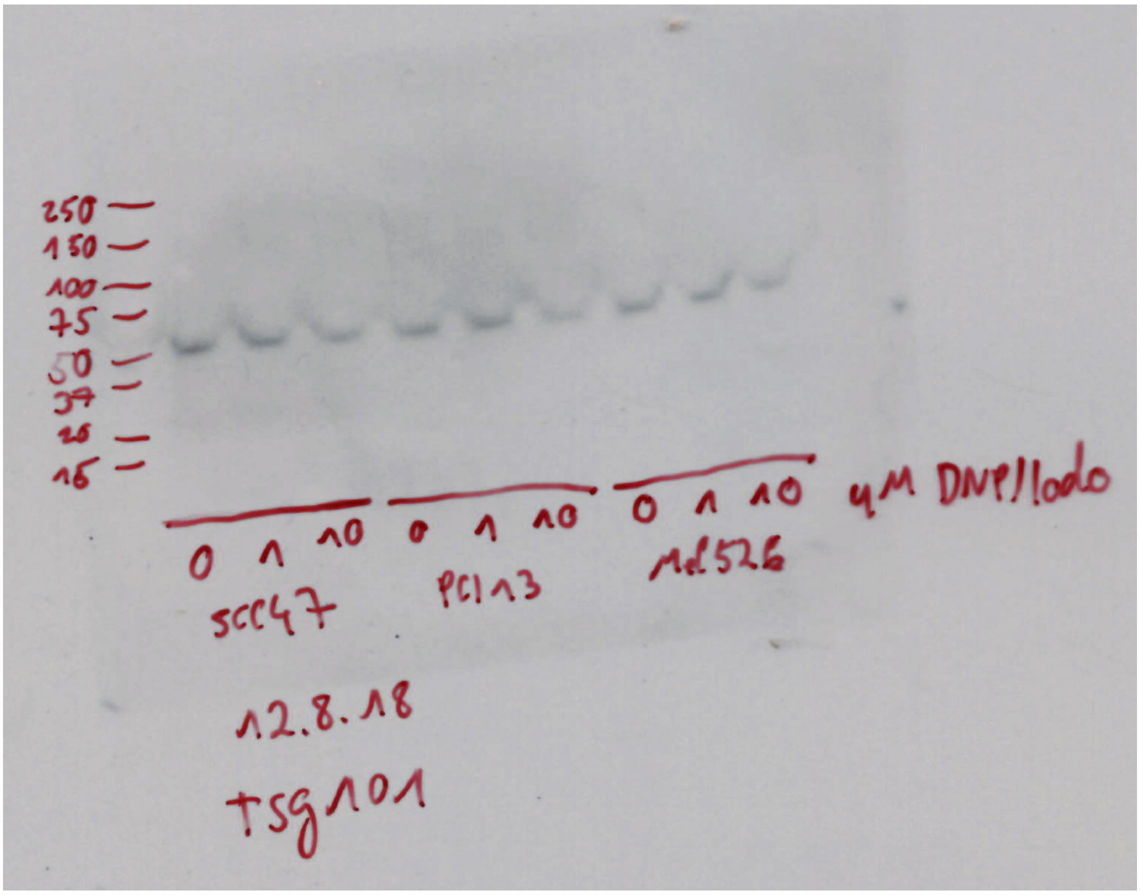

Figure S4. Full western blots of isolated UMSCC47- and PCI-13- and Mel526-derived exosomes with a TSG101 antibody. Cells were treated with indicated concentrations of IAA/DNP. Cropped version is presented in Figure 2C.

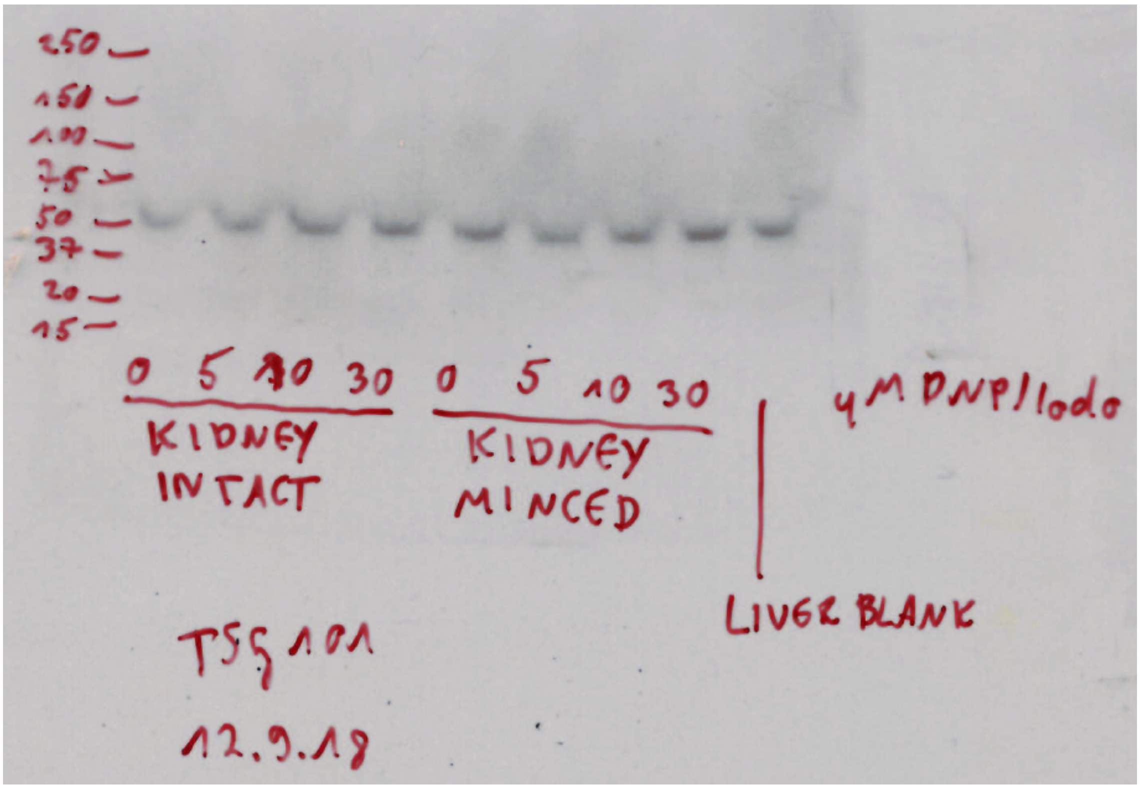

Figure S5. Full western blots of isolated exosomes derived from tissue explants with a TSG101 antibody. Explants were treated with indicated concentrations of IAA/DNP. Cropped version is presented in Figure 4D.

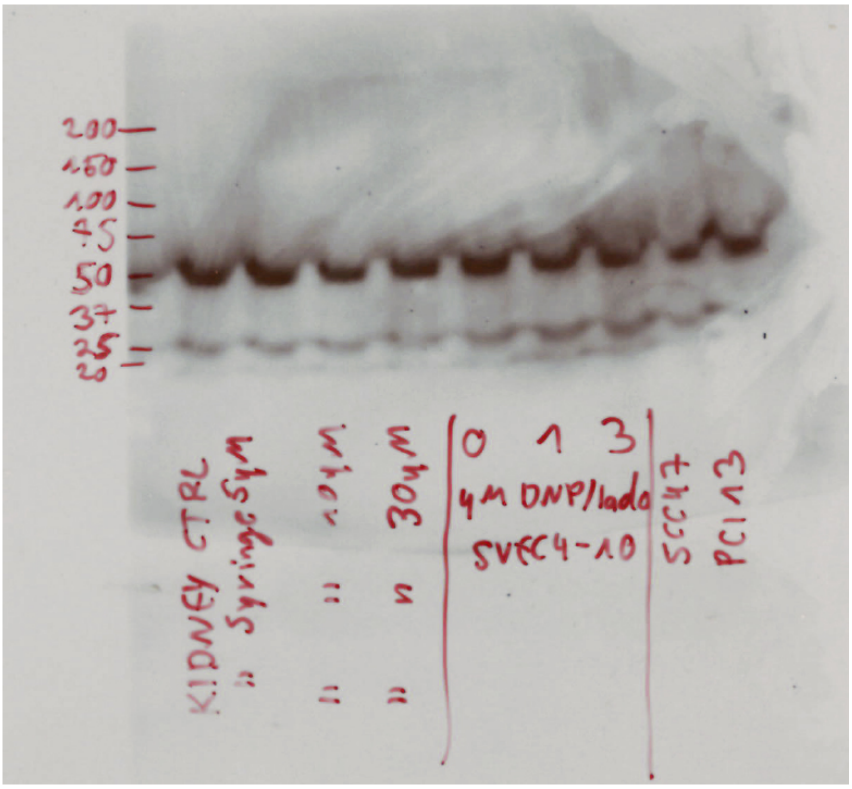

Figure S6. Full western blots of isolated exosomes derived from tissue explants with a TSG101 antibody. Explants were treated with indicated concentrations of IAA/DNP. Left 4 lanes are presented as cropped versions in Figure 4D.

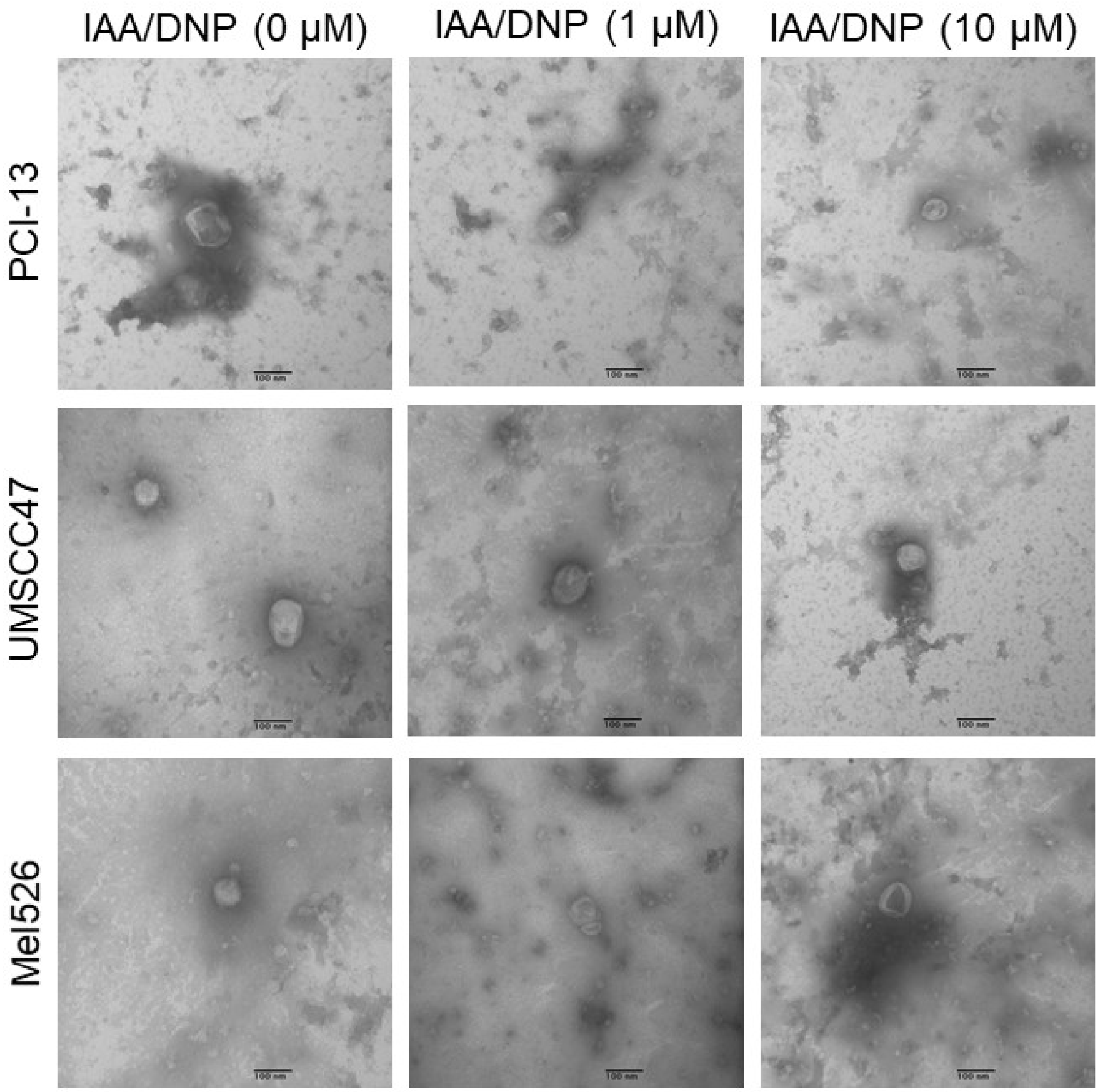

Figure S7. TEM images of isolated and negatively-stained UMSCC47-, PCI-13- and Mel526-derived exosomes. Cells were treated with indicated concentrations of IAA/DNP. Cropped versions are presented in Figure 2A.
